# Identifying the functions of two biomarkers in human oligodendrocyte progenitor cell development

**DOI:** 10.1186/s12967-021-02857-8

**Published:** 2021-05-01

**Authors:** Haipeng Zhou, Ying He, Zhaoyan Wang, Qian Wang, Caiyan Hu, Xiaohua Wang, Siliang Lu, Ke Li, Yinxiang Yang, Zuo Luan

**Affiliations:** 1 The Second Clinical College, Southern Medical University, Guangzhou, 510515 China; 2 Laboratory of Paediatrics, The Sixth Medical Centre of PLA General Hospital, Beijing, 100048 China

**Keywords:** Human oligodendrocyte progenitor cells, Biological markers, NG2, A2B5, Cell sorting

## Abstract

**Background:**

Human oligodendrocyte precursor cells (hOPCs) are an important source of myelinating cells for cell transplantation to treat demyelinating diseases. Myelin oligodendrocytes develop from migratory and proliferative hOPCs. It is well known that NG2 and A2B5 are important biological markers of hOPCs. However, the functional differences between the cell populations represented by these two biomarkers have not been well studied in depth.

**Objective:**

To study the difference between NG2 and A2B5 cells in the development of human oligodendrocyte progenitor cells.

**Methods:**

Using cell sorting technology, we obtained NG2+/−, A2B5+/− cells. Further research was then conducted via in vitro cell proliferation and migration assays, single-cell sequencing, mRNA sequencing, and cell transplantation into shiverer mice.

**Results:**

The proportion of PDGFR-α + cells in the negative cell population was higher than that in the positive cell population. The migration ability of the NG2+/−, A2B5+/− cells was inversely proportional to their myelination ability. The migration, proliferation, and myelination capacities of the negative cell population were stronger than those of the positive cell population. The ability of cell migration and proliferation of the four groups of cells from high to low was: A2B5− > NG2− > NG2+ > A2B5+. The content of PDGFR-α+ cells and the ability of cell differentiation from high to low was: NG2− > A2B5− > A2B5+ > NG2+.

**Conclusion:**

In summary, NG2+  and A2B5+ cells have poor myelination ability due to low levels of PDGFR-α+ cells. Therefore, hOPCs with a higher content of PDGFR-α+ cells may have a better effect in the cell transplantation treatment of demyelinating diseases.

**Supplementary Information:**

The online version contains supplementary material available at 10.1186/s12967-021-02857-8.

## Background

Current clinical methods for the treatment of demyelinating disease, in addition to more mature immunotherapy, focus on cell transplantation therapies. Cell transplantation is a strategy aimed at treating this disease by replacing the lost or damaged cell population [1]. The transplanted cell types consist mainly of human oligodendrocyte progenitor cells (hOPCs) and mature human oligodendrocytes (OLs). These cells may be obtained directly from foetal and adult brain tissues [2–4] or through induced embryonic stem cells (ESCs) [5, 6] or induced pluripotent stem cells (iPSCs) [7, 8]. Although many types of cells can be transplanted, their effects on the repair or regeneration of myelin post-transplantation are variable, and these effects may be associated with the different states of the transplanted cells. Therefore, it is necessary to select a cell population that is most conducive to myelin regeneration after transplantation.

Myelin oligodendrocytes are key cells for myelin formation. They are derived from migratory and proliferative hOPCs. Therefore, hOPCs are a potential option for cell transplantation for the treatment of demyelinating diseases. In addition to platelet-derived growth factor receptor alpha (PDGFR-α), which is a marker of hOPCs, chondroitin sulphate proteoglycan 4 (NG2) and A2B5 (A2B5 is an antibody that recognizes gangliosides) are two important, generally recognised markers [3, 9, 10]. Although studies have shown that both NG2-positive (NG2+) and A2B5-positive (A2B5+) cells can differentiate into oligodendrocytes in vivo and in vitro [11, 12], the differences in myelination ability between the two cell lineages have not been studied in depth. Other studies have shown that as hOPCs differentiate into mature oligodendrocytes, both of these markers are downregulated [13]. This dynamic change in expression may imply that these two markers play different roles in the development of hOPCs.

In preliminary research, we cultured extracted human foetal brain neural stem cells (NSCs) in vitro and successfully induced their differentiation into hOPCs [14]. Due to the fragile nature of hOPCs, MACSQuant^®^Tyto^®^ (Miltenyi Biotec, Bergisch-Gladbach, Germany) was chosen for cell sorting. (www.miltenyibiotec.com/local). After obtaining the target cells, we studied the gene levels of these cells. Single-cell RNA sequencing (scRNA-seq) can be used to study the different subgroups of hOPCs before sorting [15, 16], while RNA sequencing (RNA-seq) can be used to perform overall differential gene expression analysis and functional enrichment analysis on the sorted cell population [17, 18]. In vivo and in vitro cell function is assessed mainly through in vitro proliferation [19] and migration experiments [20] as well as the evaluation of the myelinating ability of the sorted cells transplanted into the shiverer mouse corpus callosum [21, 22].

In this study we investigated the differences in the proliferation, migration, and myelination ability of NG2+/− and A2B5+/− cells during the development of hOPCs, and found that the migration ability of hOPCs may be inversely proportional to their myelination ability. The results of this study provide insights into the selection of cell types for cell transplantation to treat demyelinating diseases.

## Methods

### Cultivation and identification of hOPCs

hOPCs were prepared in the Paediatric Laboratory of the Sixth Medical Centre of PLA General Hospital, China, using previously established methods of cultivation and identification. Briefly, hOPCs were induced by NSCs. The cells were cultured in a self-made medium at 37 °C in a humidified 5% CO_2_ incubator (Additional file 1: Text S1). The hOPCs were identified using immunofluorescence staining. Monoclonal mouse anti-PDGFR-α (1:250, Cat. #C2318, CST, Boston, MA, USA), rabbit anti-NG2 (1:50, Cat. #ab83178, Abcam, Cambridge, Cambridgeshire, UK) and mouse anti-A2B5 (1:50, Cat. #MAB1416, R&D Systems, Minneapolis, MN, USA) antibodies were used to identify hOPCs. Cell nuclei were stained with 4′,6-diamidino-2-phenylindole (DAPI) (1:20, Cat.#28718-90-3, Sigma-Aldrich, St. Louis, MO, USA) for 5 min and then observed using fluorescence microscopy (IX-70, Olympus Corporation, Tokyo, Japan).

### Single-cell RNA sequencing (scRNA-seq) of hOPCs

To evaluate the expression of PDGFR-α, NG2 and A2B5, scRNA-seq was performed at the Beijing Novogene Bioinformatics Technology Co (Beijing, China). We prepared a cell suspension containing a total number of cells greater than 1 × 10^6^. The prepared cell suspension was quickly loaded into the chromium microfluidic chip with 3′ chemistry, and a barcode of 10 × chromium controller was attached. The cells were then subjected to RNA reverse transcription. A sequencing library was constructed using reagents from the chromium single-cell 3′v2 kit (10 × Genomics, Pleasanton, CA, USA), according to the manufacturer’s instructions. Illumina was used for sequencing, according to the manufacturer's instructions (Illumina, San Diego, CA, USA).

### hOPC surface staining for flow cytometry and cell sorting

For the analysis of PDGFR-α, NG2 and A2B5 expression in hOPCs, the cells were digested and washed once with buffer (pH 7.2 PBS, 0.5% BSA) and centrifuged at 500×*g* for 5 min at 4 °C. Fragment crystallisable (Fc) receptors were blocked with normal mouse serum for 10 min at 25 °C. The cells were surface-stained with PDGFR-α BV421 mouse anti-human (Cat. #562799, BD Biosciences, Franklin Lake, NJ, USA), NG2 APC mouse anti-human (Cat. #FAB2585A, R&D Systems, MN, USA), or A2B5 PE mouse anti-human (Cat. #130-093-581, Miltenyi Biotec, Bergisch-Gladbach, Germany) antibodies for 30 min at 4 °C. After surface staining, hOPCs were washed once with the abovementioned buffer and centrifuged at 500×*g* for 5 min at 4 °C. The cell pellet obtained was resuspended in the same buffer for flow cytometry analysis and cell sorting.

### FlowSight image flow cytometric analysis

Cells were acquired using a FlowSight^®^ imaging flow cytometer (Amnis^®^, part of EMD Millipore, MA, USA). Cell debris and dead cells were identified using the aspect ratio and area of the cells and removed. Approximately 7000 cells were obtained during each analysis. Channel 7 was used to detect BV421, Channel 3 to acquire PE, and Channel 11 to detect APC. Single colour control samples were compensated using a compensation matrix (.rif) and converted to data analysis files (.daf) and compensated image files (.cif) using the same settings. The data were analysed using Ideas software, version 6.2.

### MACSQuant^®^Tyto^®^ cell sorting

The fluorescent anti-NG2 and anti-A2B5 antibody-labelled cells were transferred into a MACSQuant^®^Tyto^®^ Cartridge. The input sample contained 5 × 10^6^ hOPCs in 10 mL MACSQuant Tyto Running Buffer. Logical gating hierarchies were constructed using MACSQuant Tyto software before sorting. Cell debris, doublets, and dead cells were gated out, and a gate was set on the target cells. The sample was sorted at 4 mL/h using ~ 140 mbar pressure. Upon completion of sorting, negative cells were analysed using a FlowSight^®^ imaging flow cytometer to assess cell purity and yield. The expression of PDGFR-α in positive and negative cells was detected using a FlowSight^®^ imaging flow cytometer.

### RNA-seq analysis

Total RNA was isolated from the four sorted populations using RNAiso Plus (Takara Bio). RNA-seq was performed at the Beijing Novogene Bioinformatics Technology Co, using the Illumina NovaSeq platform. RNA Nano 6000 Assay Kits and the Bioanalyzer 2100 system (Agilent Technologies, CA, USA) were used to evaluate RNA integrity. A NanoPhotometer^®^ spectrophotometer (IMPLEN, CA, USA) was used to check the purity of the RNA. Clustering and sequencing (Novogene Experimental Department) were conducted according to the manufacturer’s instructions. The index-coded samples were clustered using a cBot Cluster Generation System and TruSeq PE Cluster Kit v3-cBot-HS (Illumina). Finally, the libraries were sequenced using the Illumina NovaSeq platform and 150 bp paired-end reads were generated.

### Differential expression analysis and gene ontology (GO) enrichment analysis

We used the edgeR package for the R statistical software with one scaling normalisation factor to adjust each sequenced library for read counts, and then performed differential gene expression analysis. The p values were adjusted using the Hochberg and Benjamini method. The expression level of genes was presented using fragments per kilobase of exon model per million mapped fragment (FPKM) values. FPKM of ≥ 1 indicated that the gene was expressed [23]. Differentially expressed genes were analysed for GO enrichment by the clusterProfiler R software package, and the bias in gene expression was corrected. GO terms included were as follows: biological process (BP), cellular component (CC), and molecular function (MF). When the corrected p-value was < 0.05, differentially expressed genes were considered to be significantly enriched in the GO terms.

### Cell migration assays

Cell migration was measured in Transwell filters with 8-μm pores (Corning, Tewksbury, MA, USA). Inserts were coated with human fibronectin protein (Cat. #33016015, Thermo Fisher Scientific, Waltham, MA, USA). Sorted cells (2 × 10^4^ cells), in 200 μL of medium, were loaded into the upper chambers, and 500 μL of the hOPC medium was added to the chamber. After incubation for 18 h at 37 °C, a cotton swab was used to wipe the cells that had not migrated onto the upper surface of the chamber. The migrated cells were fixed with 4% paraformaldehyde and stained with DAPI (1:20). Images were acquired using a fluorescence microscope. The migrated cells were quantified using ImageJ software [24] by analysing cell nuclei from at least five randomly selected fields per chamber. The cell migration rate was calculated as follows: cell migration rate (V1%) = N1/N2 × 100%, where *N1* refers to the initial cell seeding number, and *N2* refers to the number of migrated cells. The experiment was repeated three times independently in the laboratory.

### Cell proliferation assays

The proliferation of different cell populations was evaluated using cell counting kit-8 (CCK-8) assays (Cat. #CK04, Dojindo, Kumamoto, Japan). hOPCs (6000 cells/well) were seeded into 96-well plates and cultured for 24 h at 37 °C and 5% CO_2_. The same volume of hOPC medium was added to the blank control group, and six parallel wells were set for each group without any cells. At different experimental time points (1, 3, 5, 7, 9, and 11 days), 10 μL of CCK-8 solution was added to the wells and incubated at 37 °C for 2 h. Six wells were set for each experiment at each time point. The absorbance at 450 nm was measured with a microplate reader (BioTek, Winooski, VT, USA). At least three experiments were performed, and each was tested in triplicate. The experiment was repeated three times independently in the laboratory [25].

### Cell transplantation in shiverer mice

Homozygous shiverer mice (The Jackson Laboratory, Maine) were maintained at the Sixth Medical Centre of PLA General Hospital in a specific pathogen-free environment. All animal experiments were performed according to protocols approved by the Sixth Medical Centre of PLA General Hospital Animal Care and Use Committee (Application No. SCXK-2012-0001). Newborn pups were transplanted within a day after birth. Different cell populations (1 × 10^5^/1.5 µL) were injected bilaterally into the corpus callosum using a mouse brain stereotaxic apparatus (Stoelting, USA). At the same time, untransplanted homozygous shiverer mice (n = 6) were randomly selected as the control group. The postoperative pups were returned to their mother and were weighed every day. After weaning, each mouse was reared separately. At 3 months of age, the mice were anaesthetised with 1% pentobarbital sodium. Then mouse brain samples were perfused with PBS and fixed with 4% paraformaldehyde (Thermo Fisher Scientific, Waltham, MA, USA). The brain tissue was cut in half along the sagittal suture. The coronal section of half of the brain tissue was used for myelin basic protein immunofluorescence staining (rat anti-MBP antibody, Abcam, Cambridge, UK). A fluorescence microscope was used to observe the fluorescently labelled samples. The sagittal section of the corpus callosum of the other half of the brain tissue was used for transmission electron microscopy (TEM, H7650-B, HITACHI, Tokyo, Japan). A field of view was randomly selected for each mouse, and the remyelination rate and G-ratio were calculated as follows: remyelination rate = N1/N2, where N1 refers to the number of axons ensheathed by myelin and N2 refers to the total number of axons; G-ratio value = axonal diameter/total myelin-ensheathed fibre diameter.

### Statistical analysis

The experimental data in this study were analysed using GraphPad Prism version 8 software (GraphPad Software, San Diego, CA, USA, www.graphpad.com) and SPSS version 22.0. Differences were considered to be statistically significant at p < 0.05. Statistical significance was determined using independent samples Student’s t-tests or one-way ANOVA followed by Bonferroni tests. All experimental data are expressed as mean ± SD.

## Results

### hOPC identification and biomarker detection

**Fig. 1:**
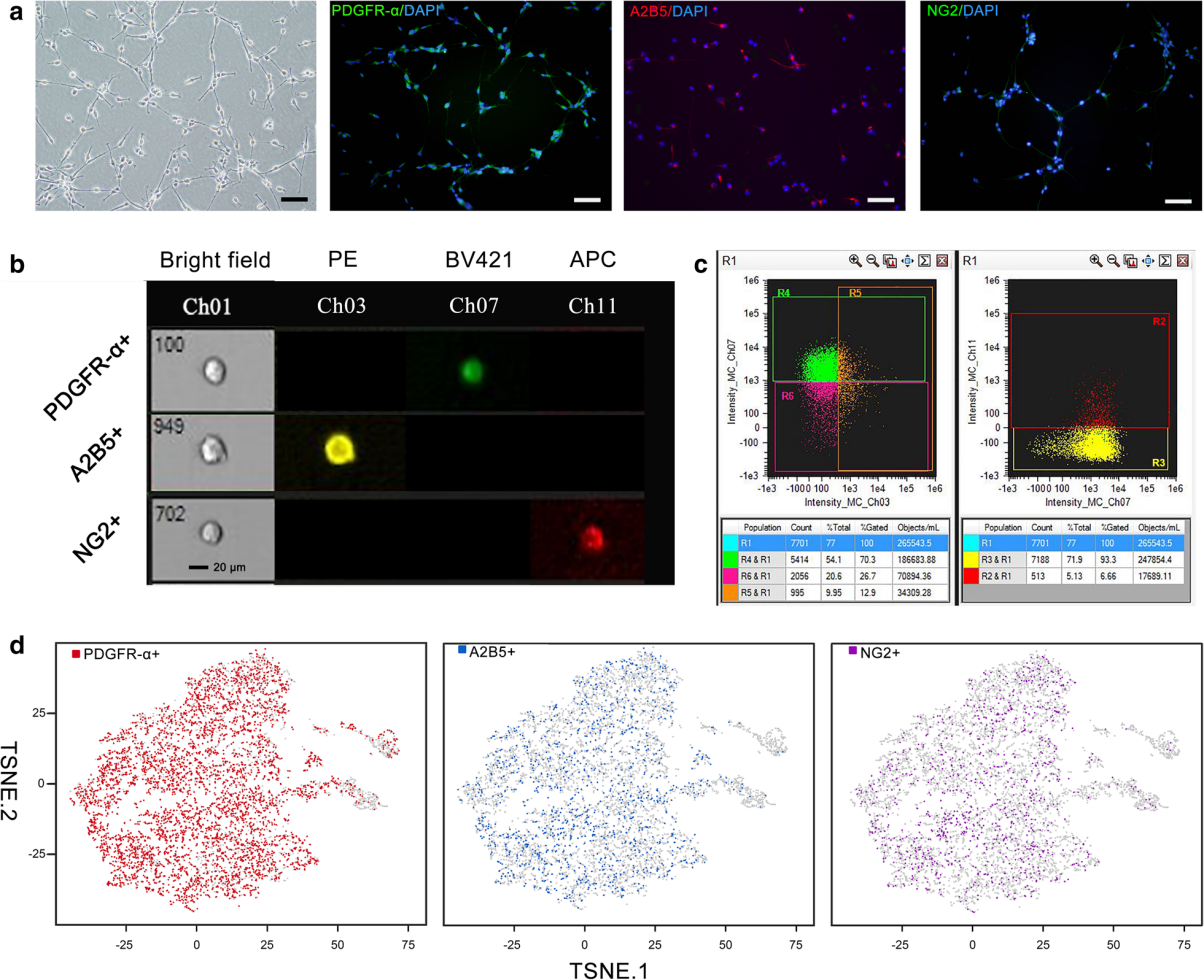
Identification of hOPC markers. **a** Three markers of hOPCs were identified using cell immunofluorescence staining: PDGFR-α, A2B5, and NG2. Scale bar is 200 μm. **b** Images of single cells detached in the presence of PDGFR-α+, A2B5+, and NG2+ cells, as observed using flow cytometry. Ch01: Bright field, Ch03: A2B5-PE, Ch07: PDGFR-α-BV421, Ch11: NG2-APC. Scale bar is 20 μm. **c** Gating strategy to define PDGFR-α/A2B5/NG2 cells. R4: PDGFR-α+ cells, R5: A2B5+ cells, R2: NG2+ cells. **d** scRNA-seq of hOPCs. Maps of t-SNE of 7885 cells from high-dimensional images of hOPCs coloured according to the cell type. *hOPC* human oligodendrocyte progenitor cells, *PDGFR-α* platelet-derived growth factor receptor alpha, *scRNA-seq* single-cell RNA sequencing

We identified hOPCs using cell morphology and immunofluorescence staining. hOPCs had a typical bipolar, bead-like appearance, and expressed PDGFR-α, NG2, and A2B5 (Fig. 1a). Based on the results of flow cytometry analysis, the proportion of PDGFR-α+ cells was 74.13 ± 1.74% of the whole cell population; A2B5+ cells made up 25.97 ± 0.74%; NG2+  cells comprised 14.41 ± 0.81% (Fig. 1b). Volume uniformity of hOPCs, which had a cell diameter < 20 µm, and the expression of the three cell markers on a single-cell membrane were observed (Fig. 1c). scRNA-seq showed that the proportions of PDGFR-α+, A2B5+ and NG2+ cells in hOPCs were 67% (n = 5309), 16% (n = 1243) and 13% (n = 1002), respectively (Fig. 1d).

### hOPC sorting and PDGFR-α expression in positive and negative cells

**Fig. 2:**
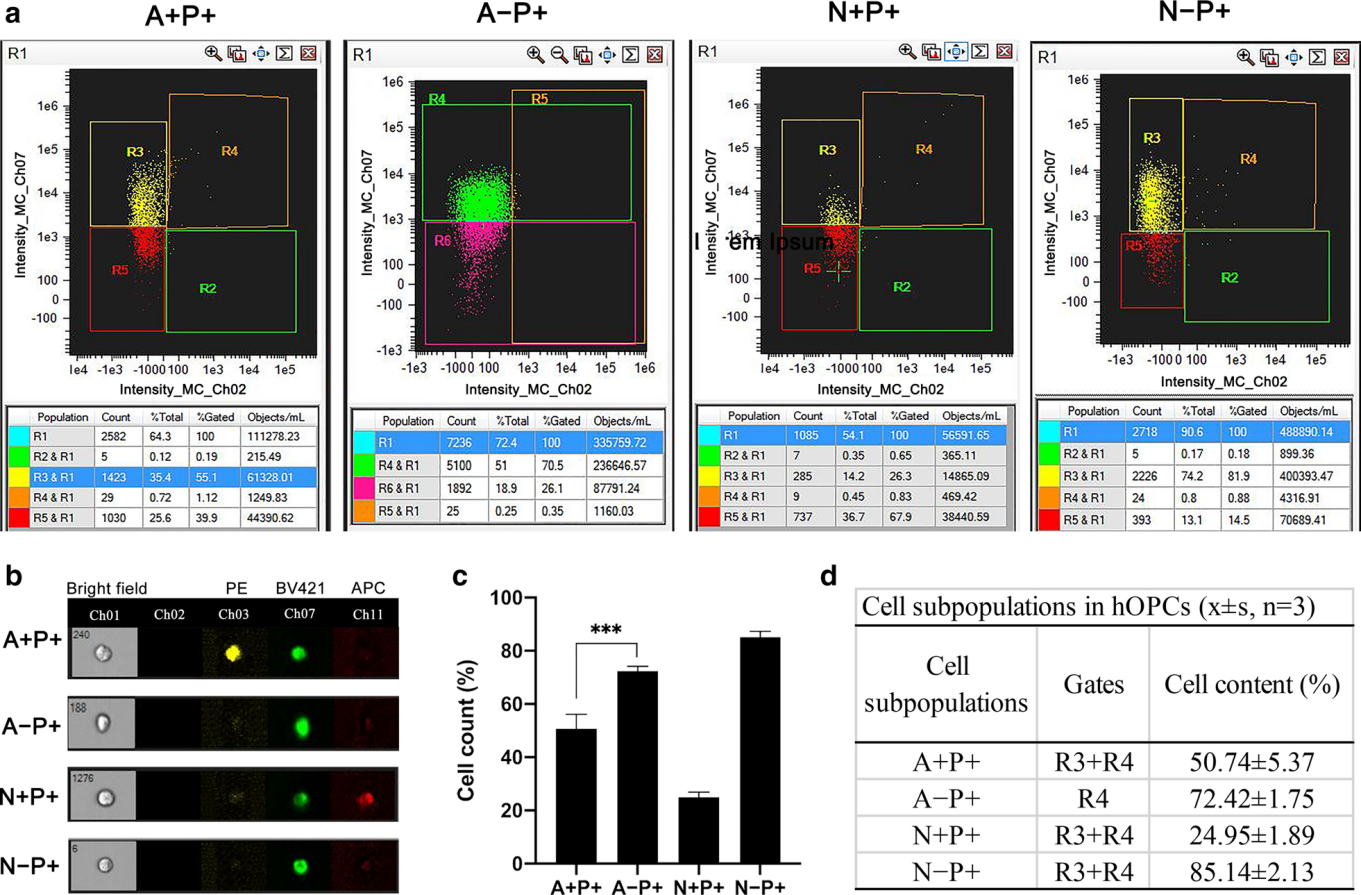
The expression of PDGFR-α in positive and negative cells. **a** Gating strategy to define PDGFR-α+ cells in A2B5+/− and NG2+/− populations. **b** Images of single cells detached in the presence of A + P +, A − P +, N + P +, and N − P + cells. **c**, **d** Cell counts of different cell populations. Bars represent the mean. Error bars show the standard error of the mean. A + P + , PDGFR-α+ cells in A2B5+ cells; A − P + , PDGFR-α+ cells in A2B5− cells; N + P + , PDGFR-α+ cells in NG2+ cells; N − P + , PDGFR-α+ cells in NG2− cells

To obtain NG2+/− and A2B5+/− cells, we performed cell sorting on hOPCs. After sorting, the viability of the sorted cells was above 98%. In the negative group, the proportion of A2B5+ cells dropped from 28.1 to 4.03%, and the proportion of NG2+ cells decreased from 8.22 to 1.01%. NG2+ and A2B5+ cells were almost completely absent in the NG2− and A2B5− cell populations (Additional file 3: Fig. S1). Next, we detected the expression of PDGFR-α in NG2+/− and A2B5+/− cells. The results showed that PDGFR-α+ cells were present in the four groups of cells (Fig. 2a, b). The level of PDGFR-α+ in NG2− cells was the highest, reaching 85 ± 2.13%, while that in NG2+ cells was the lowest, reaching only 24.95 ± 1.89%. The difference between the groups was statistically significant (** ~ ****, Fig. 2c, d).

### RNA-seq for the proliferation, migration, and myelination ability

**Fig. 3:**
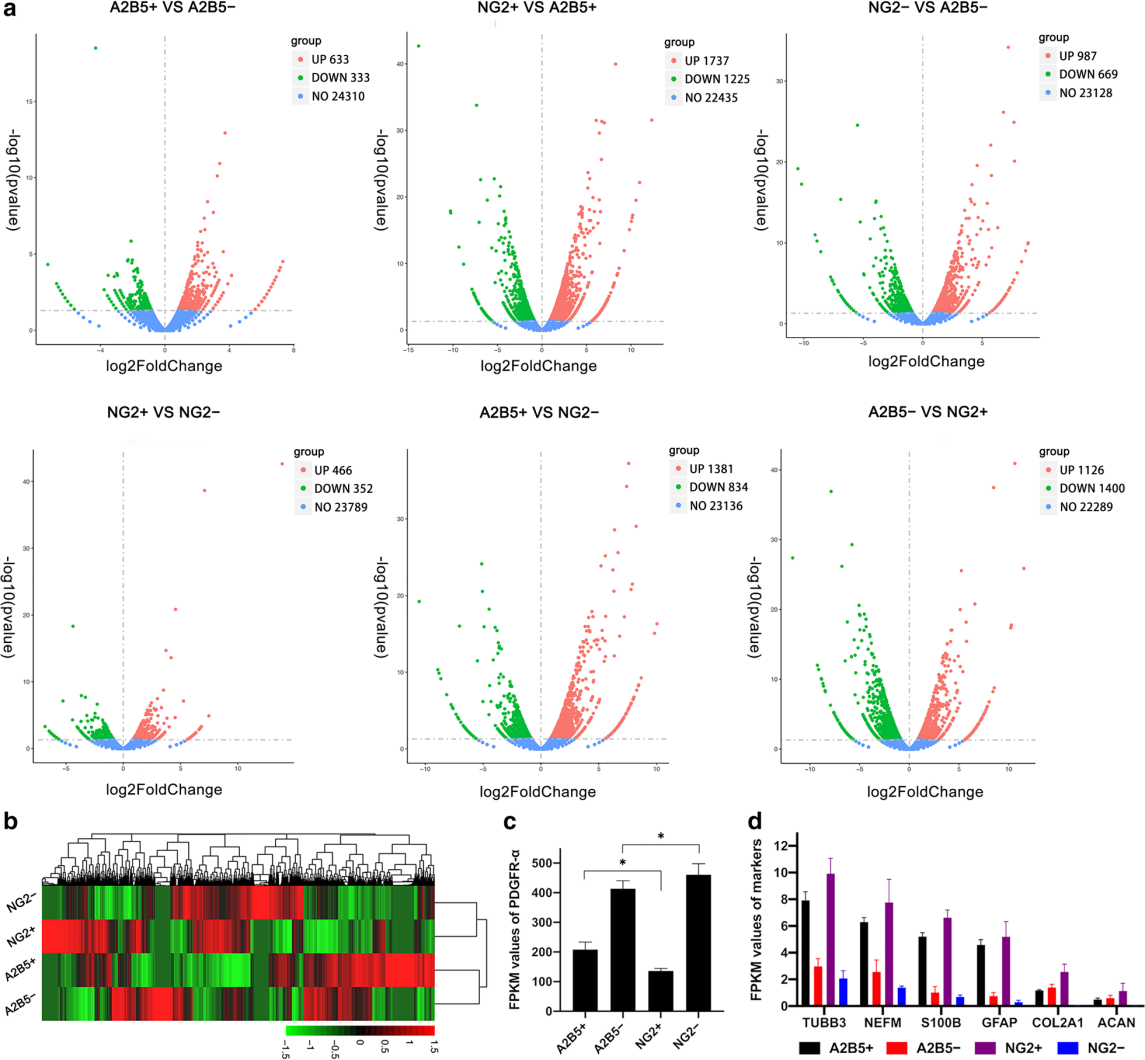
Differential expression analysis. **a** Volcano plot illustrating differentially regulated gene expression between the four groups of cells. Red dots indicate upregulated genes, and blue dots indicate downregulated genes. Values are presented as the log2 of tag counts. **b** Heat map showing the relative gene expression in NG2+/− and A2B5+/− cells. Red and green indicate upregulated and downregulated genes, respectively. **c**, **d** Graph showing the FPKM value of markers in different cell populations. Bars represent the mean. Error bars show the standard error of the mean. *FPKM* fragments per kilobase of exon model per million mapped fragments

In order to study the differential gene expression of the four groups, we performed RNA-seq. The results showed that, compared with A2B5+ cells, NG2+ cells had 1737 upregulated and 1225 downregulated genes. Compared with A2B5− cells, A2B5+ cells had 633 upregulated and 333 downregulated genes. Compared with A2B5+ cells, NG2− cells had 1381 upregulated and 834 downregulated genes. Compared with A2B5− cells, NG2− cells had 967 upregulated and 669 downregulated genes. Compared with NG2− cells, NG2+ cells had 466 upregulated and 352 downregulated genes. Compared with NG2+ cells, A2B5− cells had 1126 upregulated and 1400 downregulated genes (Fig. 3a). Figure 3b shows a heat map of the genes which were differentially expressed among the four cell populations.

Next, we detected the expression of the main markers of OPCs, neurons, astrocytes, chondrocytes, and OLs in these four groups of cells. The results showed that in NG2+ and A2B5+ cells, in addition to the expression of NG2 and A2B5, PDGFR-α was also expressed. More importantly, PDGFR-α was abundantly expressed in NG2− and A2B5− cells. Further analysis showed that among the four groups of cells, PDGFR-α expression was the highest in NG2− cells and the lowest in NG2+ (* ~ ****, Fig. 3c). In addition, among these four groups of cells, the expression of neuron markers TUBB3 and NEFM, astrocyte markers S100β and GFAP, and chondrocyte markers COL2A1 and ACAN was much lower than that of PDGFR-α (****, p < 0.0001). In addition, the expression of neuron and astrocyte markers in the positive cell population was higher than that in the negative cell population (****, p < 0.0001), but there was no difference between NG2+ and A2B5+ cells, as well as NG2− and A2B5− cells. There was no difference in the expression of chondrocyte markers among these four groups (Fig. 3d). However, the main OL lineage markers were not expressed in these four groups of cells. These markers include GALC, PLP1, APC, CNP, MBP, MOG, and MAG (Additional file 2: Table S1).

**Fig. 4:**
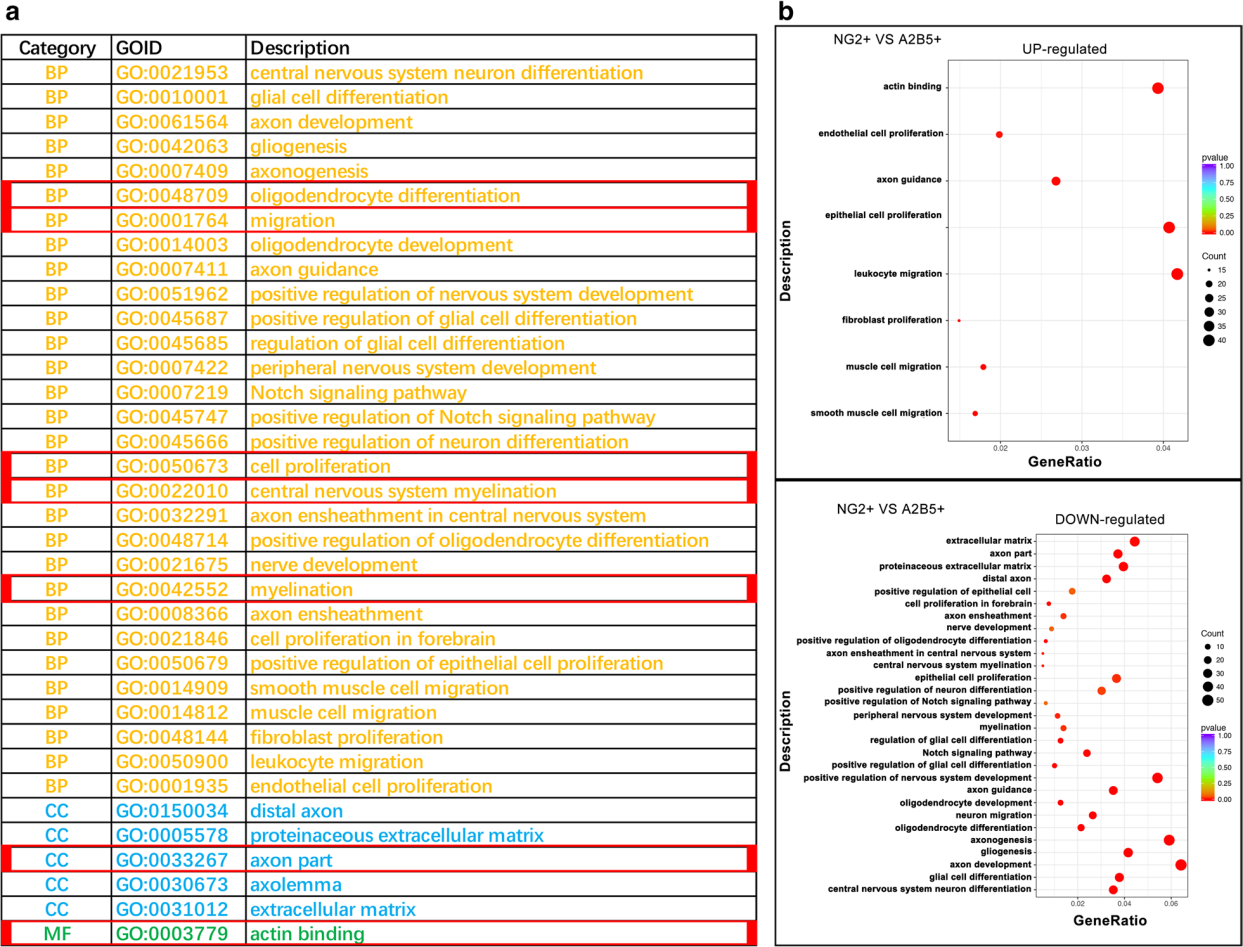
GO enrichment analysis. **a** Table with specific instances of BP, CC, and MF. **b** Bubble plots of the main enriched GO terms (NG2+ vs A2B5+) for the upregulated and downregulated genes. *GO* gene ontology

We then performed GO enrichment analysis on the upregulated and downregulated genes in each group. Figure 4a shows the key GO terms. BP mainly included cell proliferation (GO:0050673), migration (GO:0050673), oligodendrocyte differentiation (GO:0048709), and myelination (GO:0042552); CC mainly included extracellular matrix (GO:0031012) and axon part (GO:0033267); MF included actin-binding (GO:0003779). Bubble plots showed that in NG2+ and A2B5+ cells, the upregulated genes were mainly involved in cell migration and proliferation, while the downregulated genes were mainly involved in oligodendrocyte differentiation (Fig. 4b). The results of the other groups are provided in Additional file 4: Fig S2. In terms of cell migration and proliferation, the gene enrichment of the four groups of cells from high to low was A2B5− > NG2− > NG2+ > A2B5+. The gene enrichment intensity for oligodendrocyte differentiation from high to low was NG2− > A2B5− > A2B5+ > NG2+.

### Evaluation of the migration and proliferation function of hOPCs in vitro

**Fig. 5:**
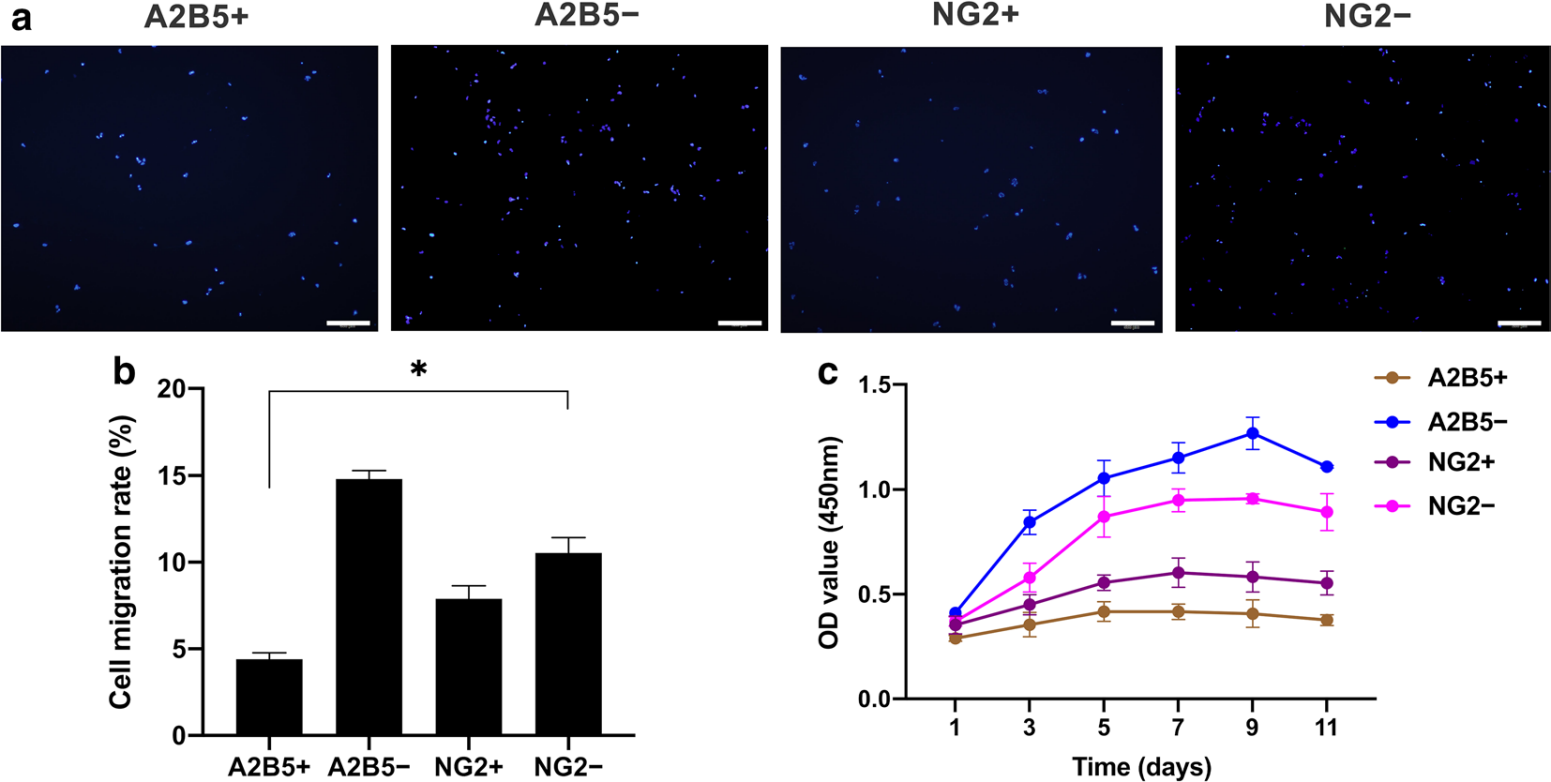
Evaluation of the in vitro migration and proliferation function of hOPCs. **a** Migration assay. Microscopic images show DAPI staining of migrating cell nuclei. Scale bar is 200 μm. **b** Graph showing the cell migration rate. Bars represent the mean. Error bars show the standard error of the mean. **c** CCK-8 cell proliferation assay after 1, 3, 5, 7, 9, and 11 days. *hOPC* human oligodendrocyte progenitor cells, *CCK* cell counting kit

We used Transwell assays to evaluate the migration of the cells. After the cells had migrated for 18 h, the nuclei were stained with DAPI (Fig. 5a). The migration rates of A2B5+ and A2B5− cell populations were 4.41 ± 0.38% and 14.82 ± 0.48%, respectively. The migration rates of NG2+ and NG2− cell populations were 7.89 ± 0.75% and 10.04 ± 0.18%, respectively. Statistical analysis showed that the migration of NG2+ was stronger than that of A2B5+ (*, p = 0.0278) and that the migration of NG2− was weaker than that of A2B5− (*, p = 0.0267). The migration of A2B5+ was weaker than that of A2B5− (*, p = 0.0240), and the migration of NG2+ was weaker than that of NG2− (**, p = 0.0017) (Fig. 5b). The proliferation assay results showed that the proliferation of NG2+ was stronger than that of A2B5+ (**, p = 0.0015), while the proliferation of NG2− was weaker than that of A2B5− (**, p = 0.0028). The proliferation of A2B5− was stronger than that of A2B5+ (**, p = 0.0027), and the proliferation of NG2+ was weaker than that of NG2− (**, p = 0.0079) (Fig. 5c).

### hOPCs were transplanted into the corpus callosum of shiverer mice

**Fig. 6:**
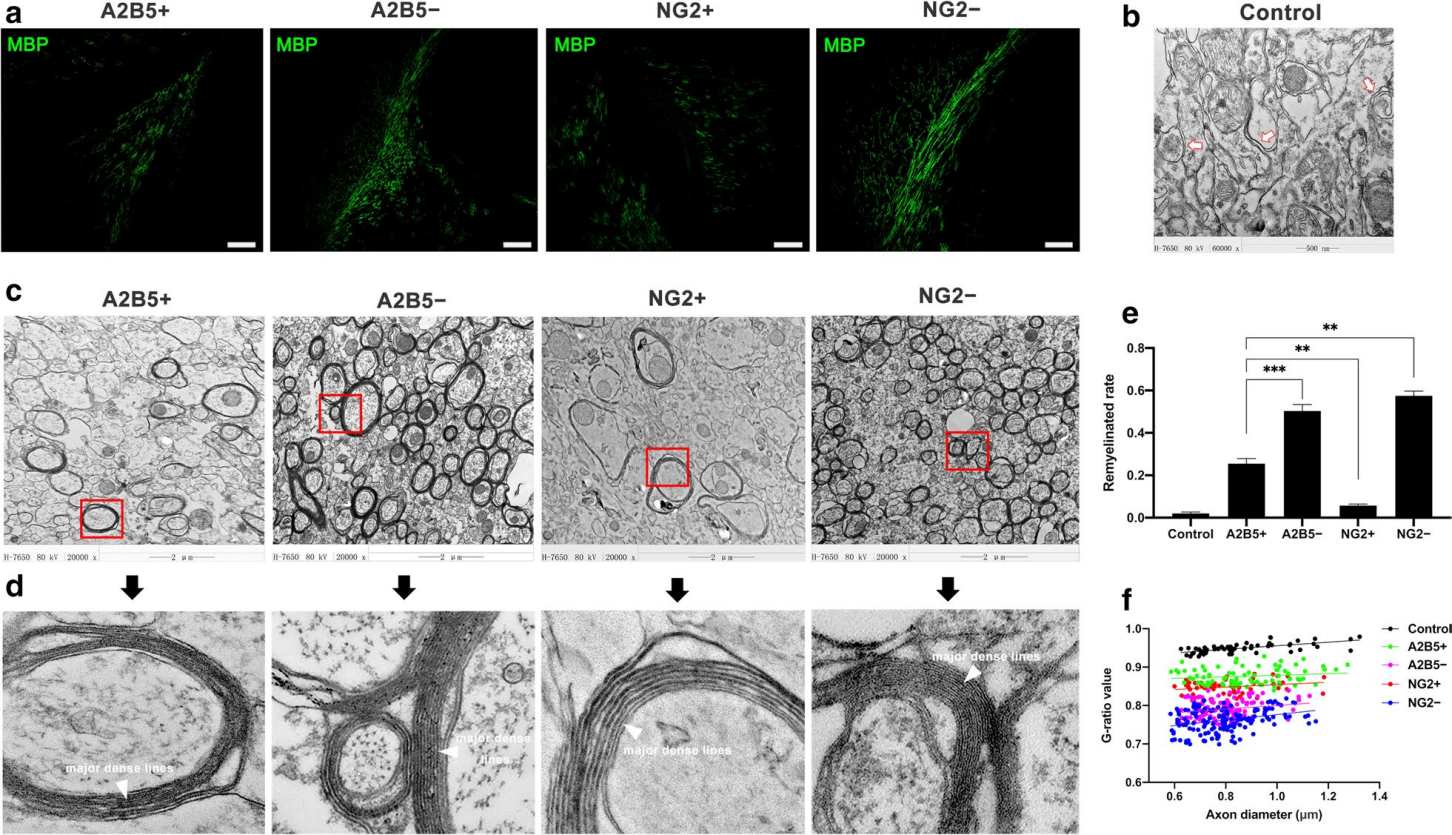
TEM and immunohistochemical analysis. **a** Alexa 488-labelled, donor-derived MBP (green) of four transplanted cells. Scale bar is 200 μm. **b**, **c** Electron micrographs showing the sagittal section through the corpus callosum of untreated (**b**, magnification: 60,000 ×) and transplanted (**c**, magnification: 20,000 ×) shiverer mice. **d** Higher magnification of the area indicated by the red box in **c**. **e** The graph showing the remyelination rate of different groups and **f** the plot showing the G-ratio value. Bars represent the mean. Error bars show the standard error of the mean. *TEM* transmission electron microscopy, *MBP* myelin basic protein

We evaluated the myelinating ability of these four groups of cells in vivo. The mature myelin is characterised by the expression of MBP. Therefore, MBP immunofluorescence staining was performed on shiverer mouse brain tissue to confirm the presence of mature myelin. There was filamentous MBP expression in the corpus callosum of transplanted mice (Fig. 6a). In addition, MBP can condense consecutive layers of myelin together; therefore, we used electron microscopy to assess myelin condensation. Untreated shiverer mice axons typically had no myelin ensheathment or single loose wrapping of uncondensed myelin, such that major dense lines did not form (Fig. 6b, arrowhead). The number of myelin sheaths in the corpus callosum of A2B5− and NG2− cells was more than that of A2B5+ and NG2+ cells (Fig. 6c). Although the four groups of axons were wrapped in multiple layers of compact myelin, the major dense lines of the negative cell groups were more obvious than those of the positive cell groups (Fig. 6d, arrowhead). The remyelination rate and G-ratio analyses of the four groups of cells showed that NG2− cells exhibited the strongest myelination effect, while NG2+ cells had the weakest effect (* ~ ****, Fig. 6e, f).

## Discussion

Both NG2+ and A2B5+ cells can differentiate into oligodendrocytes in vivo and in vitro [26, 27] and form myelin structures in brain tissue, consistent with our experimental results. Other studies on the two cell populations also compared their myelinating ability. According to some reports, A2B5+ cells have a greater capacity to ensheath nanofibers, and NG2+ cells fail to differentiate into oligodendrocytes as quickly as A2B5+ cells [28]. Our in vivo experiments also showed that the myelinating effect of NG2+ cells is not as notable as that of A2B5+ cells.

The reason for this phenomenon may be that NG2+ cells are multifunctional cells with lineage plasticity, and NG2+ cells contain heterogeneous progenitor cells with different differentiation potentials [29]. As per our mRNA-seq results, the expression of the main markers of neurons and astrocytes in NG2+ cells was higher than that in A2B5+ cells. Moreover, the degree of GO enrichment related to proliferation and migration in NG2+ cells was also higher than that in A2B5+ cells. Because transplanted NG2+ cells migrate extensively in the brain and differentiate into oligodendrocytes, astrocytes, and even neurons, the rates and effects of myelination of NG2+ cells are inferior to those observed for A2B5+ cells after cell transplantation. In addition, flow cytometry and mRNA-seq results showed that the PDGFR-α content in A2B5+ cells was higher than that in NG2+ cells. PDGFR-α is considered to be the main contributor to OPCs myelination [30]; therefore, the difference in the PDGFR-α content between the two groups of cells might be one of the reasons for the difference in their myelination ability.

We also found that the proliferation, migration, and myelination ability of the negative cell population was stronger than that of the positive cell population. In addition, NG2− cells showed the strongest myelinating ability, while NG2+ cells showed the weakest myelinating ability. A2B5− cells showed the strongest proliferation and migration, while A2B5+ cells showed the weakest proliferation and migration. Our flow cytometry results revealed that the proportion of PDGFR-α+ cells in the negative cell population was higher than that of the positive cells. The proportion of PDGFR-α+ cells in NG2− cells was as high as 85%, while that of NG2+ cells was only 25%. Similarly, mRNA-seq results also showed that PDGFR-α was highly expressed in the negative population. In addition, genes with GO terms related to proliferation, migration, and myelination were also highly expressed in the negative population. We also tested the markers associated with OLs using mRNA-seq. However, we found that OL lineage markers were not expressed in the four groups of cells. This showed that our cell line mainly contained OPCs that had not yet differentiated into OLs. Moreover, the four populations of cells did not contain OL cell populations, so the difference in the functions of these four populations of cells was not due to OLs. We speculated that the reason why the negative cell population showed excellent proliferation, migration, and myelination ability was mainly due to a large number of PDGFR-α+ cells.

## Conclusion

In summary, we used cell sorting to sort self-made hOPCs and obtained a large number of sterile, high-purity NG2+/− and A2B5+/− cells with high viability. The feasibility of using this technology to sort relatively fragile cells was verified in this study, which establishes the value of the new sorting technology for future clinical cell transplantation processes. Through genetic testing and cell function research, we clarified that the migration, proliferation, and myelination of a single-cell population are weaker than those of a mixed cell population, and that cell migration is inversely proportional to myelination ability. By comparing the differences between the functions of NG2+/− and A2B5+/− cells during the development of hOPCs into OLs, we propose that hOPCs with a high ratio of PDGFR+ cells may be the most suitable option for cell transplantation.

## Supplementary Information

**Additional file 1: Text S1.** The preparation of the hOPCs.

**Additional file 2: Table S1**. Cell markers in the NG2+/− and A2B5+/− cells.

**Additional file 3: Figure S1.** MACSQuant^®^Tyto^®^ cell sorting.

**Additional file 4: Figure S2.** GO analysis.

## Data Availability

The data used to support the findings of this study are available from the corresponding author upon request.

## Abbreviations

ANOVA: Analysis of variance
BP: Biological process
CCK-8: Cell counting kit-8
ECM: Extracellular matrix
GO: Gene ontology
hOPCs: Human oligodendrocyte progenitor cells
OLs: Human oligodendrocytes
MBP: Myelin basic protein
NSCs: Neural stem cells
NG2: Chondroitin sulphate proteoglycan 4
PDGFR-α: Platelet-derived growth factor receptor alpha
RNA-seq: RNA sequencing
scRNA-seq: Single-cell RNA sequencing
TEM: Transmission electron microscopy
