# Supplementary material for: Identifying the functions of two biomarkers in human oligodendrocyte progenitor cell development
Source: J Transl Med. 2021 May 1;19:188. doi: 10.1186/s12967-021-02857-8 (PMC8088696; doi:10.1186/s12967-021-02857-8)
Supplement: Supplementary file 1 — Additional file 1: Text S1. The preparation of the hOPCs. [file 12967_2021_2857_MOESM1_ESM.docx]

**Text S1: The preparation of the hOPCs**

Human fetal central nervous system (CNS) tissues obtained from 10- to 13-week-old embryos were provided by the Pediatric Laboratory of the Sixth Medical Center of the Chinese People’s Liberation Army General Hospital. All women consented to donate the aborted fetuses. The tissue was prepared as a single cell suspension. Primary cells were cultured at a density of 1 × 10^6^ cells/mL in primary culture medium containing DMEM/F12 (Cat. #C11330500BT, Gibco), 1% L-glutamine (Cat. #10888-022, Gibco), 1% N2 supplement (Cat. #17502-048, Gibco), 2% B27 supplement (Cat. #17504-044, Gibco), 20 ng/mL bFGF (Cat. #AF-100-18B, PeproTech), 20 ng/mL EGF (Cat. #AF-100-15, PeproTech), 5 μg/mL heparin (Cat. #H3149, Sigma), and 1% penicillin/streptomycin (Cat. #15140, Invitrogen). Every 3–4 days, two-thirds of the medium was removed and replaced with fresh primary culture medium. The expanded cells formed “neurospheres” after 5–7 days of culture. Neurospheres were repeatedly blown 40 to 50 times to obtain single-cell suspensions. The single-cell suspensions were cultured in primary culture medium but without B27 supplement for 7 days. On the seventh day, two-thirds of the medium was removed and replaced with fresh OPC medium. OPC medium was prepared by adding 2% B27 supplement, 20 ng/mL bFGF, 1% penicillin/streptomycin, 5 μg/mL heparin, and 2 mM L-glutamine to Neurobasal-A medium. After 7–10 days of proliferation, the OPCs were induced successfully. Cells are cultured in 6-well plates with a cell seeding volume of 2 × 10^5^ per well. Every 3–4 days, two-thirds of the medium was removed and replaced with fresh OPC medium. After 6 days of proliferation, the OPCs reached 80–90% confluence. At this time, the cells were digested and subcultured.
