# Supplementary material for: Identifying the functions of two biomarkers in human oligodendrocyte progenitor cell development
Source: J Transl Med. 2021 May 1;19:188. doi: 10.1186/s12967-021-02857-8 (PMC8088696; doi:10.1186/s12967-021-02857-8)
Supplement: Supplementary file 2 — Additional file 2: Table S1. Cell markers in the NG2+/− and A2B5+/− cells. [file 12967_2021_2857_MOESM2_ESM.docx]

**Table S1**: Cell markers in the NG2+/− and A2B5+/− cells ($\bar{x}\pm s$)

| Cell Markers | FPKM values (n=3) | | | |
| --- | --- | --- | --- | --- |
|  | NG2+ | NG2− | A2B5+ | A2B5− |
| PDGFR-α | 134.67±5.51 | 456.54±34.03 | 211.76±2.94 | 415.72±28.96 |
| A2B5 | 39.03±0.93 | 102.05±0.41 | 557.26±0.31 | 0.57±0.89 |
| NG2 | 637.09±0.52 | 0.35±0.98 | 30.37±0.74 | 35.64±0.24 |
| TUBB3 | 9.92±1.24 | 2.07±0.28 | 7.21±0.53 | 3.97±0.31 |
| NEFM | 7.76±0.93 | 1.38±0.14 | 6.93±0.64 | 2.23±0.34 |
| S100B | 6.62±0.59 | 0.72±0.17 | 5.77±0.36 | 0.85±0.55 |
| GFAP | 5.19±0.68 | 0.26±0.43 | 4.35±0.47 | 0.65±0.72 |
| COL2A1 | 2.56±0.46 | 0.03±0.61 | 1.26±0.53 | 1.33±0.29 |
| ACAN | 1.13±0.23 | 0.01±0.22 | 0.49±0.42 | 0.57±0.74 |
| GALC | 0 | 0 | 0 | 0 |
| PLP1 | 0 | 0 | 0 | 0 |
| APC | 0 | 0 | 0 | 0 |
| CNP | 0 | 0 | 0 | 0 |
| MBP | 0 | 0 | 0 | 0 |
| MOG | 0 | 0 | 0 | 0 |
| MAG | 0 | 0 | 0 | 0 |

FPKM, Fragments Per Kilobase of exon model per Million mapped fragments. NG2+, NG2-positive cell populations. NG2−, NG2-negative cell populations. A2B5+, A2B5-positive cell populations. A2B5−, A2B5-negative cell populations.
