# Supplementary material for: Identifying the functions of two biomarkers in human oligodendrocyte progenitor cell development
Source: J Transl Med. 2021 May 1;19:188. doi: 10.1186/s12967-021-02857-8 (PMC8088696; doi:10.1186/s12967-021-02857-8)
Supplement: Supplementary file 3 — Additional file 3: Figure S1. MACSQuant®Tyto® cell sorting. [file 12967_2021_2857_MOESM3_ESM.docx]

**
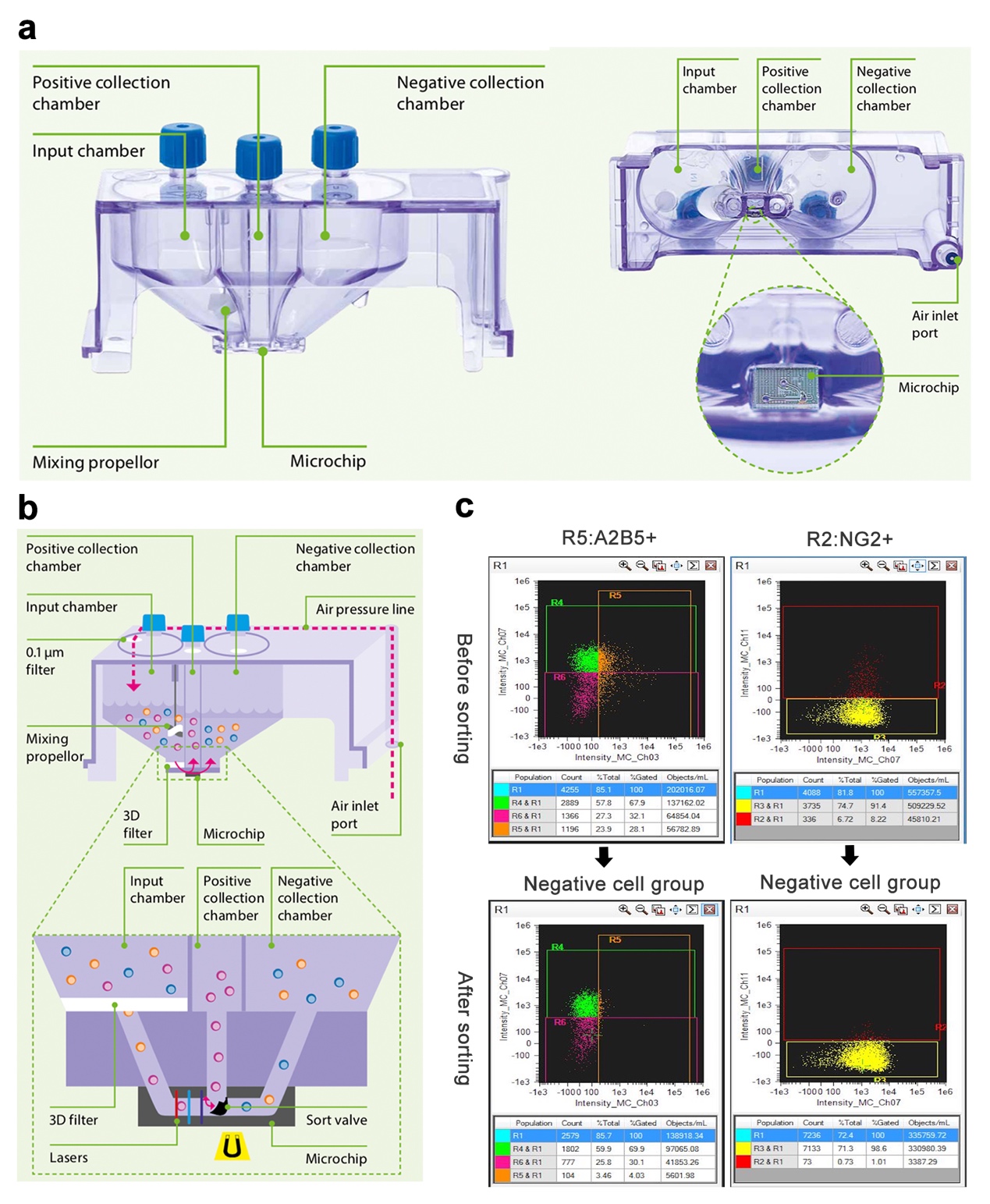
**

**Fig S1.** MACSQuant®Tyto® cell sorting. **a** MACSQuant®Tyto® Cartridge and the bottom view of the Cartridge with a zoomed-in image of the microchip. **b** Sorting mechanism of MACSQuant®Tyto®. **c** Flow cytometry using the FlowSight® imaging flow cytometer used to evaluate the sorting effect of A2B5+ and NG2+ cells. In the negative cell group, the proportion of A2B5+ cells dropped from 28.1 to 4.03%, and the proportion of NG2+ cells decreased from 8.22 to 1.01%.
