# Supplementary material for: Identifying the functions of two biomarkers in human oligodendrocyte progenitor cell development
Source: J Transl Med. 2021 May 1;19:188. doi: 10.1186/s12967-021-02857-8 (PMC8088696; doi:10.1186/s12967-021-02857-8)
Supplement: Supplementary file 4 — Additional file 4: Figure S2. GO analysis. [file 12967_2021_2857_MOESM4_ESM.docx]

**
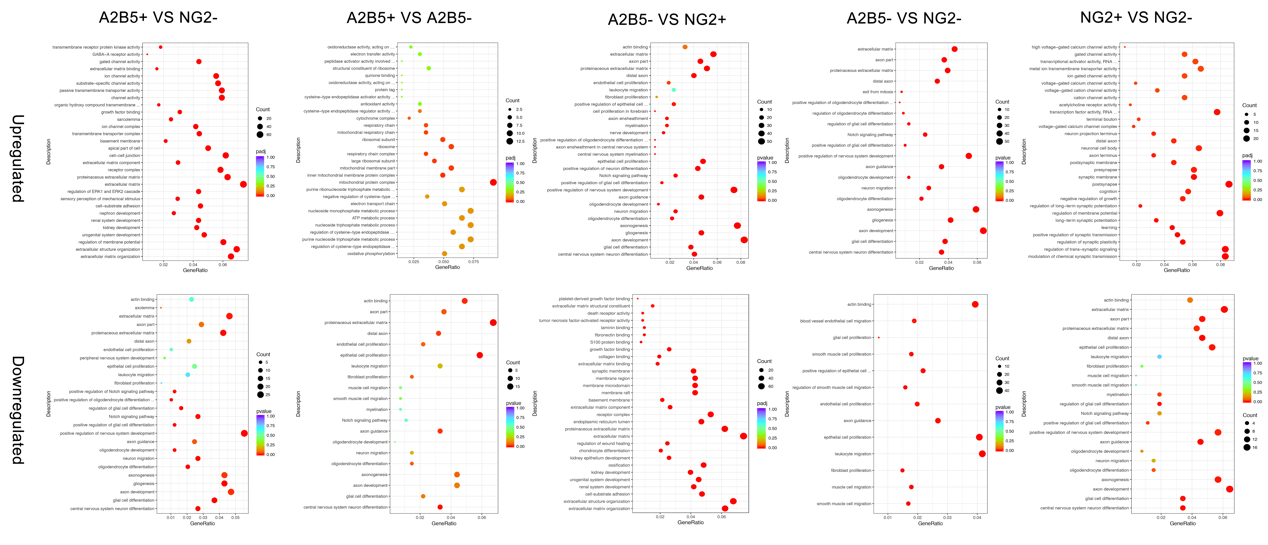
**

**Fig S2.** GO analysis. Bubble plots showing the main enriched GO terms for the upregulated and downregulated genes.

GO, gene ontology.
